# Oxidation of heat shock protein 60 and protein disulfide isomerase activates ERK and migration of human hepatocellular carcinoma HepG2

**DOI:** 10.18632/oncotarget.7093

**Published:** 2016-01-31

**Authors:** Chung-Yi Lin, Chi-Tan Hu, Chuan-Chu Cheng, Ming-Che Lee, Siou-Mei Pan, Teng-Yi Lin, Wen-Sheng Wu

**Affiliations:** ^1^ Institute of Medical Sciences, Tzu Chi University, Hualien, Taiwan; ^2^ Division of Gastroenterology, Department of Internal medicine Taichung Tzu Chi Hospital, Buddhist Tzu Chi Medical Foundation, Hualien, Taiwan; ^3^ Research Centre for Hepatology, Department of Internal Medicine, Buddhist Tzu Chi General Hospital and Tzu Chi University, Hualien, Taiwan; ^4^ Department of Laboratory Medicine and Biotechnology, College of Medicine, Tzu Chi University, Hualien, Taiwan; ^5^ Department of Surgery, Buddhist Tzu Chi General Hospital, Hualien, School of Medicine, Tzu Chi University, Hualien, Taiwan

**Keywords:** hepatocyte growth factor, hepatocellular carcinoma, reactive oxygen species, extracellular signal-regulated kinases, heat shock protein 60

## Abstract

Hepatocyte growth factor (HGF) and its receptor c-Met were frequently deregulated in hepatocellular carcinoma (HCC). Signaling pathways activated by HGF-c-Met are promising targets for preventing HCC progression. HGF can induce the reactive oxygen species (ROS) signaling for cell adhesion, migration and invasion of tumors including HCC. On the other hand, extracellular signal-regulated kinases (ERK), member of mitogen activated kinase, can be activated by ROS for a lot of cellular processes. As expected, HGF-induced phosphorylation of ERK and progression of HCC cell HepG2 were suppressed by ROS scavengers. By *N*-(biotinoyl)-*N*′-(iodoacetyl)-ethylenediamine (BIAM) labeling method, a lot of cysteine (−SH)-containing proteins with M.W. 50–75 kD were decreased in HepG2 treated with HGF or two other ROS generators, 12-O-tetradecanoyl-phorbol-13-acetate (TPA) and phenazine methosulfate. These redox sensitive proteins were identified by matrix-assisted laser desorption ionization-time of flight mass spectrometry. Among them, two chaperones, heat shock protein 60 (HSP60) and protein disulfide isomerase (PDI), were found to be the most common redox sensitive proteins in responding to all three agonists. Affinity blot of BIAM-labeled, immunoprecipitated HSP60 and PDI verified that HGF can decrease the cysteine (−SH) containing HSP60 and PDI. On the other hand, HGF and TPA increased cysteinyl glutathione-containing HSP60, consistent with the decrease of cysteine (−SH)-containing HSP60. Moreover, depletion of HSP60 and PDI or expression of dominant negative mutant of HSP60 with alteration of Cys, effectively prevented HGF-induced ERK phosphorylation and HepG2 migration.

In conclusion, the redox sensitive HSP60 and PDI are required for HGF-induced ROS signaling and potential targets for preventing HCC progressions.

## INTRODUCTION

Hepatocellular carcinoma (HCC) ranks sixth in incidence and third in mortality among all cancers worldwide [1]. Although metastatic spreads are responsible for poor prognosis of most HCCs [2, 3], effective target therapy aiming at the signaling pathway leading to tumor metastasis of HCCs has not been fully established thus far.

Tumor metastasis occurs via complicated processes, including epithelial mesenchymal transition (EMT), migration and invasion of primary tumor, followed by intravasation, extravasation and colonization at the metastatic loci [4]. Within tumor microenvironment, the primary tumors may activate stromal and inflammatory cells leading to the secretion of a lot of metastatic factors including hepatocyte growth factor (HGF), epidermal growth factor (EGF) [4–7], and transforming growth factor-β (TGFβ) [8]. Among the metastatic factors, HGF, a well-known scatter factor, was frequently elevated during the progression of cancers including HCC. The receptor tyrosine kinase of HGF, c-Met, is deregulated in HCCs which were closely associated with early HCC recurrence [9]. Patients with high c-Met expressing HCCs usually have shorter 5-year survival after curative resection than those with low c-Met expressing HCCs [9–12]. Also, the effects of HGF on metastatic changes of HCCs including EMT, migration and invasion have been observed [13–15]. Therefore, HGF-c-Met signaling is now regarded as one of the promising therapeutic targets for prevention of HCC progression [16–19]. However, the side effects caused by c-Met inhibitors including neutropenia and liver and bone marrow toxicity [20, 21] were frequently observed, probably due to the ubiquitous c-Met expression and its essential physiological functions [21]. Taken together, it is urgent to search more suitable downstream effectors of c-Met as therapeutic targets for prevention of HCC progression.

Recently, the role of reactive oxygen species (ROS) in signal transductions that trigger tumor progression was highlighted [22–25]. ROS including superoxide radical (·O_2_^−^), hydrogen peroxide (H_2_O_2_) and reactive hydroxyl radicals (·OH) are constantly generated in aerobic organisms during intracellular metabolism and in response to environmental stimuli. The redox switch of different types of cysteine-thiols in critical signaling proteins highlights as a reversible means to regulate their signal transducing activity, analogous to phosphorylation [26]. Aberrant ROS signaling may result in many physiological and pathological changes [26] such as cell cycle progression [27] apoptosis, aging [28], and diabetic complication [29]. Importantly, ROS signaling can be induced by a lot of metastatic factors [for reviews, 30–32] such as HGF [33–35], TGFβ [36, 37] and EGF [38] to trigger cell adhesion, migration and invasion of tumors including stomach cancer, colon cancer and HCC. In addition, hypoxia [39], the tumor promoter 12-O-tetradecanoyl-phorbol-13-acetate (TPA) [40, 41] and integrin engagement [41, 42] may also induce ROS signaling for tumor progression.

Among the intracellular signal components, mitogen activated protein kinase (MAPK) family including extracellular signal-regulated kinases (ERK) and c-jun N-terminal kinase were known to be activated by ROS for triggering a lot of cellular processes [34, 35, for reviews, 43–46]. Moreover, activation of ROS-MAPK signal cascades was closely associated with migration and invasion of a lot of tumor cells including breast cancer [47] melanoma [48] and HCC [40, 41].

The detailed mechanisms for ROS to trigger MAPK signaling have been intensively studied. ROS can activate MAPK *via* oxidative inactivation of a lot of proteins that have negative regulatory effects on signal transduction [for reviews, 26, 49, 50]. For example, inhibition of protein tyrosine phosphatases by H_2_O_2_ contributes to the induction of distinct MAPK activation profiles [51]. On the other hand, ROS can directly activate signaling kinases, such as epidermal growth factor receptor [52] and apoptosis signal-regulating kinase 1 [53], to potentiate MAPK signaling.

In this report, two redox sensitive chaperones, heat shock protein 60 (HSP60) and protein disulfide isomerase (PDI), were found to be required for HGF-induced ERK activation and HCC migration. The cysteine residue on HSP60 involved in these events was also identified.

## RESULTS

### Involvement of ROS signal in HGF-induced tumor progression of HCC

**Figure 1:**
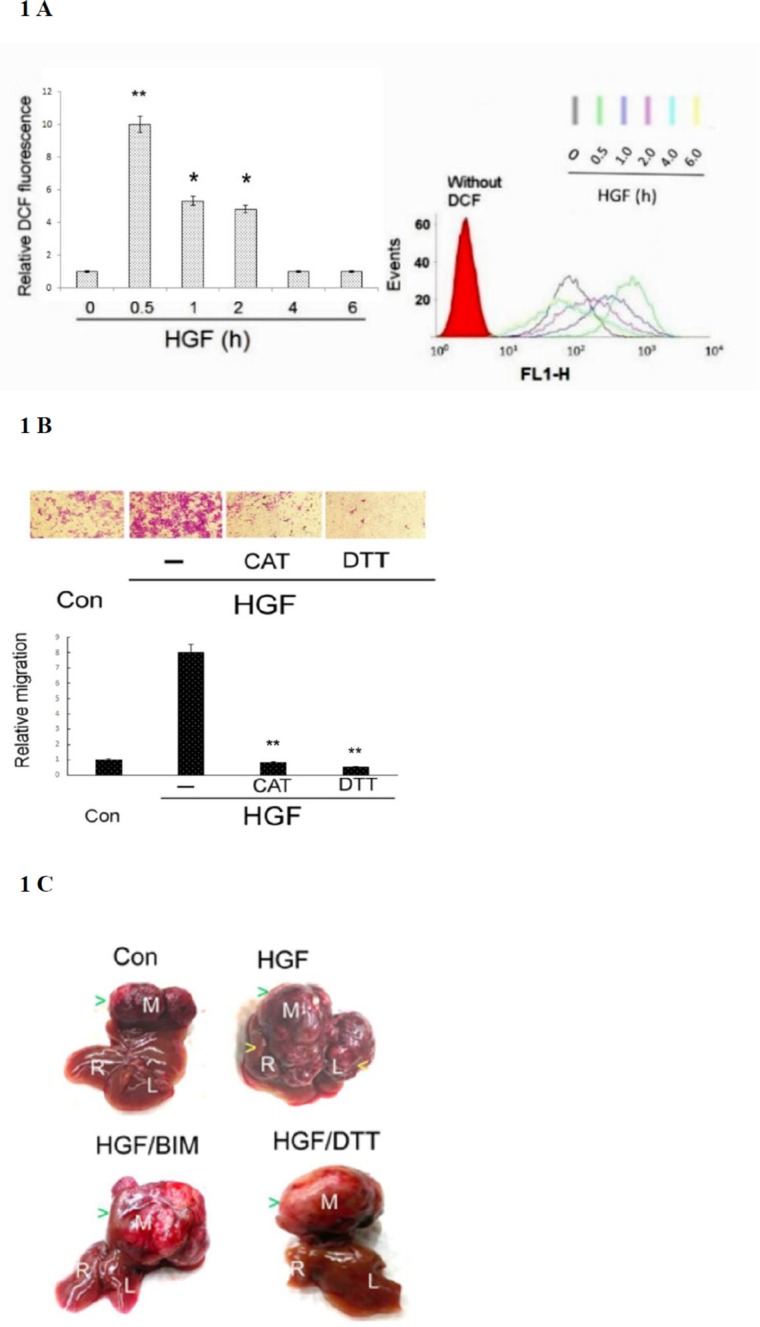
HGF-induced ROS generation was required for tumor progression of HepG2 (**A**) HepG2 were treated with 25 nM HGF as indicated times. Cells were then labeled with DCF-DA for 30 min followed by flow cytometric analysis. The acquisition data and merged profile of the fluorescent distribution for oxidized DCF (events *vs* FL1-H) were shown with different colors representing the DCF fluorescence at various time points (upper panel). Red area under the left curve represented the cells without DCF-DA labeling. The quantitative figure for the area under the curves for different time point is demonstrated on left panel. Relative fluorescent intensities of DCF were estimated, taking the data of time zero sample as 1.0. (**) and (*) represent statistical significance (Student's *t* test: *p* < 0.005 and *p* < 0.05, respectively; *N* = 3) for differences between the indicated samples and time zero group. (**B**) HepG2 cells on migration culture insert were untreated (Con) or treated with 25 nM HGF or HGF plus indicated inhibitor for 48 h. Transwell migration assay were performed. Pictures were taken under phase contrast microscope (200 *×* magnification) (upper panel) and the migrated cells were quantitated (lower panel). Relative migratory activity were estimated, taking the data of time zero sample as 1.0. (**) represent statistical significance (Student's *t* test: *p* < 0.005, *N* = 3) for differences of relative migratory activity between the indicated samples and the HGF-only group. CAT: catalase, DTT: dithiotheritol (**C**) HepG2 cells were directly inoculated into the middle liver lobe of SCID mice, followed by intraperitoneal administration of HGF with or without indicated inhibitors twice a week. The mice were sacrificed after 3 months, and their livers were photographed. The letters in white (M, R, and L) represent middle, right, and left liver lobes, respectively. The green arrow heads indicate the positions of primary tumors located in middle lobes of each treatment whereas yellow arrow heads indicate the secondary tumors in left/right liver lobes of the HGF-only sample. The data is representative of three reproducible experimental groups. BIM: Bisindolylmaleimides. DTT: dithiotheritol.

In our previous studies, we demonstrated TPA induced sustained ROS generation in HepG2 by DCF-DA labeling coupled with flow cytometry [40]. Using the same method, we also found 25 nM HGF induced ROS generation by 10-fold in HepG2 within 30 min, declined to 5–6 fold during 1–2 h and returned to basal level after 4–6 h (Figure 1A). Whether ROS generation was required for HepG2 progression induced by HGF was investigated using ROS scavengers. As demonstrated in Figure 1B, HGF induced HepG2 cell migration by 8-fold, which was abolished by the H_2_O_2_ degrading enzyme catalase (CAT) (500 units/ml) and SH-containing anti-oxidant dithiotheritol (DTT) (0.5 mM), indicating that ROS was required for HGF-induced HepG2 cell migration. Whether ROS was involved in intrahepatic metastasis (I.M.) of HepG2 triggered by HGF was further investigated in severe combined immunodeficiency (SCID) mice established previously [54]. HepG2 was directly injected into the middle liver lobe of SCID mice, followed by intraperitoneal administration of Dulbecco's Modified Eagle Medium (DMEM) (vehicle), vehicle-containing HGF (1.6 ng/g mouse) or HGF (1.6 ng/g mouse) coupled with the antioxidant DTT (50 μmole/g mouse) or Bisindolylmaleimides (BIM) (0.5 μmole/g mouse). BIM is an inhibitor of protein kinase C (PKC) which was essential for mediating HGF-induced migration, invasion and metastasis of HepG2 [54]. As shown in Figure 1C, a large tumor was observed in the middle liver lobe three months after the inoculation of HepG2 in vehicle (medium)-treated mice; however, no metastatic tumor developed in the left and right lobes (SPSS test : *p* < 0.005, *N* = 3). In contrast, both primary and metastatic tumors developed in whole liver of mice injected with HGF (SPSS test: *p* < 0.005, *N* = 3). This showed the capability of HGF in promoting I.M. of HCC. Moreover, HGF induced-I.M. of HepG2 was prominently suppressed by DTT and BIM (SPSS test: *p* < 0.005, *N* = 3). Thus, ROS is required for HGF-induced I.M. of HepG2 in SCID mice.

### HGF induced ERK phosphorylation in a ROS-dependent manner

**Figure 2:**
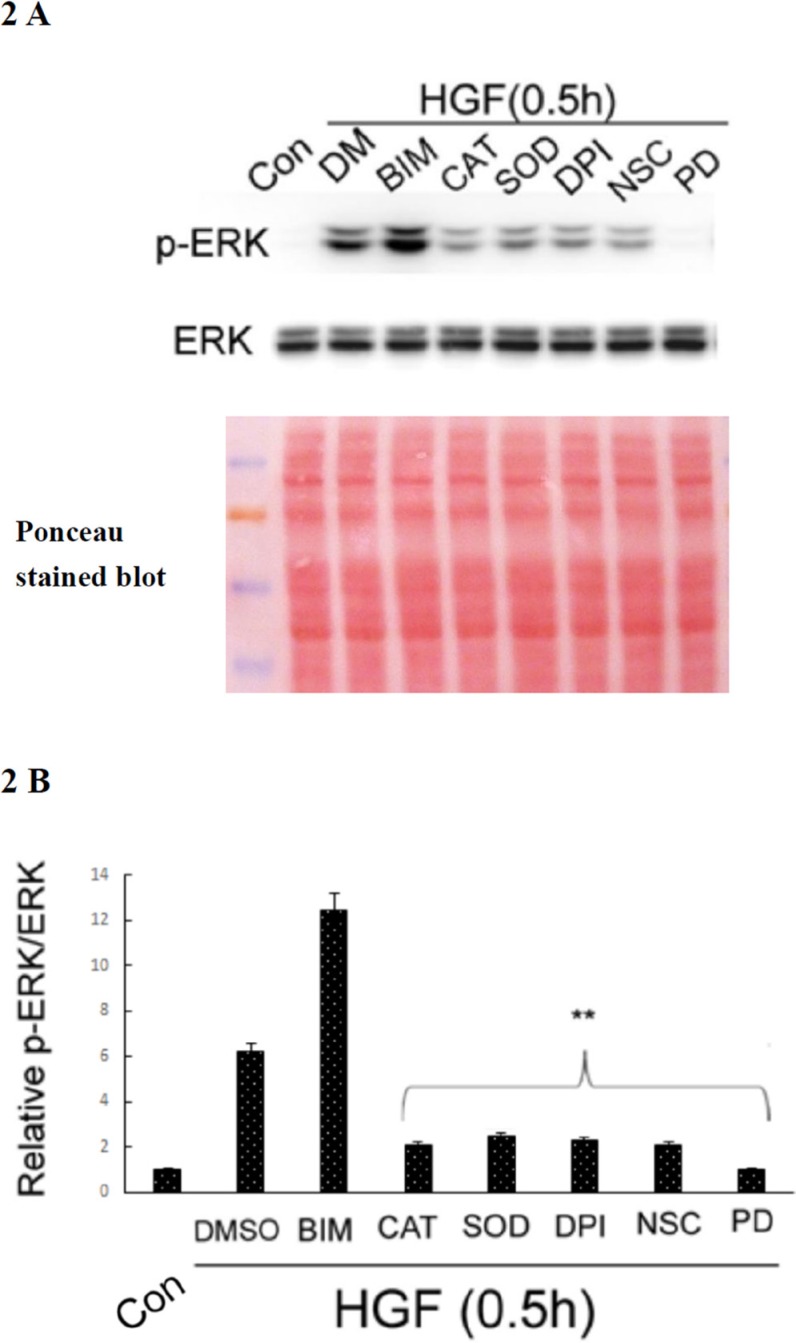
HGF induced ROS-dependent ERK phosphorylation HepG2 were untreated (Con), treated with HGF alone or HGF coupled with the indicated inhibitors for 0.5 h. DMSO (DM) was used as a solvent control. Western blots of phosphorylated ERK (p-ERK) was performed, using ERK as the internal control. Ponceau stained blot was also shown as loading control. **(A) (B)** is the quantitative figure for (A). Relative intensities of p-ERK was estimated, taking the data of untreated cells as 1.0. (**), (^##^) represent statistical significance (Student's *t p* < 0.005; *n* = 3) for intensity differences between the (HGF/inhibitor) and (HGF/DMSO) groups. BIM: Bisindolylmaleimides, CAT: catalase, SOD: superoxide dismutase (SOD), DPI: diphenyleneiodonium chloride, NSC: NSC23766, PD: PD98059.

Activation of ERK was involved in a lot of cellular processes including HCC cell migration induced by metastatic factors such as EGF [77]. Moreover, we have demonstrated that ERK was downstream of ROS in the signaling pathway triggered by TPA, leading to migration and invasion of HepG2 [40, 41]; whether it was the same in HGF-treated HepG2 was further examined. As demonstrated in Figure 2A and 2B, HGF-induced ERK phosphorylation at 30 min was dramatically suppressed (by about 90–93%) not only by CAT (500 units/ml) and the superoxide degradation enzyme, superoxide dismutase (SOD) (500 units/ml), but also by diphenyleneiodonium (DPI) (20 nM) and NSC23766 (NSC) (150 μM), inhibitors of NAPDH oxidase and RAC-1GTPase, respectively. Notably, RAC-1 is a well-known regulator of NAPDH oxidase required for ROS generation [55, 56]. This result indicated that HGF induced ERK phosphorylation in a ROS-dependent manner in HepG2. As a positive control, PD98059 (7.0 μM), the inhibitor of MEK which is the upstream kinase of ERK, totally suppressed HGF-induced ERK phosphorylation. On the other hand, HGF-induced ERK phosphorylation was enhanced by the PKC inhibitor BIM (5.0 μM) (Figure 2A and 2B). This is consistent with the previous finding that PKC plays as a negative regulator of ERK, required for HGF-induced fluctuant ERK signaling for focal adhesion turnover and cell migration of HepG2 [54].

### Detection of redox sensitive proteins in HepG2 treated by HGF and other ROS generators

**Figure 3:**
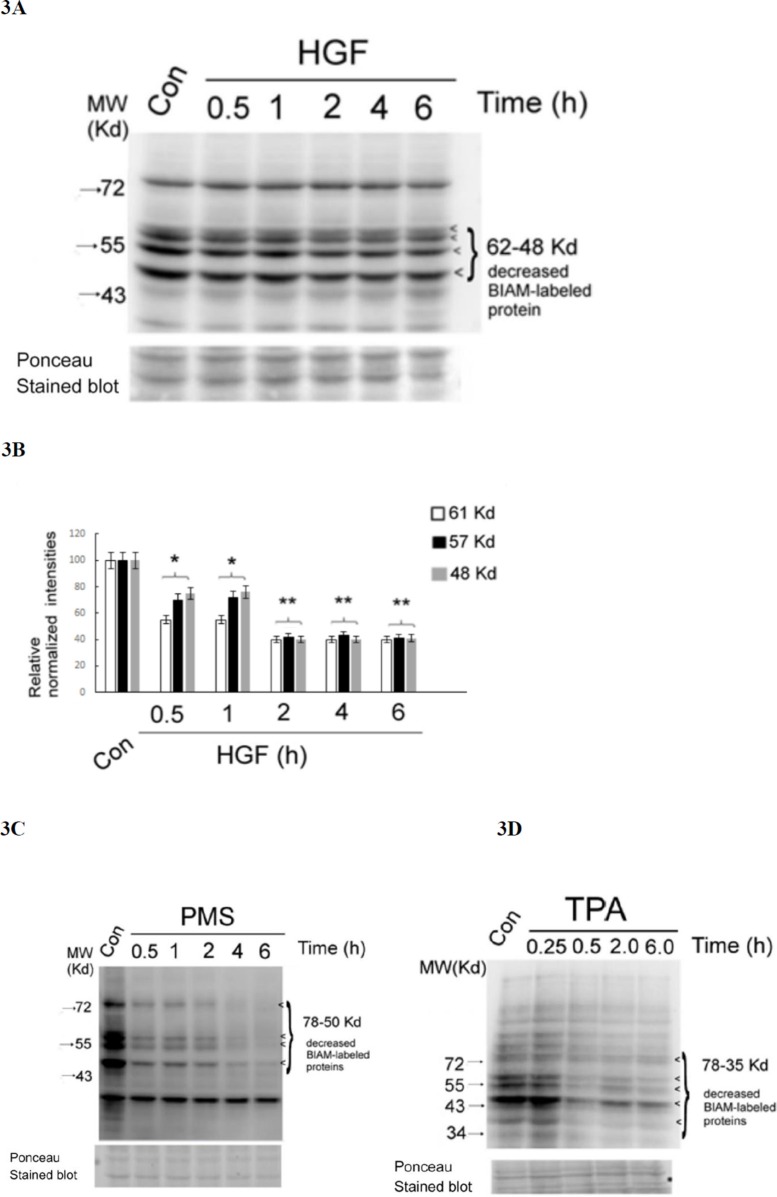
HGF and other ROS generators decreased multiple SH-containing proteins in HepG2 HepG2 cells were untreated (Con), treated with 25 nM HGF **(A)**, 50 nM TPA (C) or 20 nM PMS (D) for indicated times. Affinity blots of BIAM-labeled proteins were performed using avidin-HRP. The position of BIAM-labeled proteins decreased in the treated samples were indicated by arrow heads. Ponceau stained blots were shown for normalizing the band intensities of redox sensitive proteins. **(B)** is the quantitative figure for (A). Relative intensities for the normalized redox sensitive proteins with indicated M.W. was estimated, taking the data of the untreated cells as 100. (**) and (*) represent statistical significance (Student's *t* test: *p* < 0.005 and *p* < 0.05, respectively; *N* = 3) for differences of the proteins with specific M.W. between the indicated sample and the untreated cells. (**C**) and (**D**) are representatives of two reproducible experiments.

In proteins, the thiol group (−SH) of cysteine residue is particularly sensitive to oxidation. To explore the critical ROS targets essential for signal transduction induced by HGF, the thiol-modifying reagents *N*-(biotinoyl)-*N*'-(iodoacetyl)-ethylenediamine (BIAM) [57, 58] was employed. Protein oxidation can be detected by loss of reactivity of thiols (SH-) containing proteins with BIAM using affinity blot visualized by avidin–linked horseradish peroxidase (HRP). As demonstrated in Figure 3A and 3B, several SH-containing proteins with molecular weight (M.W.) from 50 to 61 Kd significantly decreased by 25–50% after HGF treatment for 0.5 h, further decreased by 60% at 2 h, and sustained until 6 h. This suggested that HGF can trigger significant oxidation of SH-containing proteins within the M.W. range as indicated. In parallel, the tumor promoter TPA (50 nM), a potent ROS generator in HepG2 [40, 41], induced a very dramatic decrease of SH-containing proteins with more broad M.W. range (from 35 to 78 Kd) at 30 min and sustained until 6 h (Figure 3C). As a positive control, treatment of HepG2 with the H_2_O_2_ generation agent phenazine methosulfate (PMS) (20 nM) for 0.5–2 h also greatly decreased (by about 90%) the SH-containing proteins with M.W. range of 78–50 Kd, which further disappeared after 4–6 h (Figure 3D).

**Figure 4:**
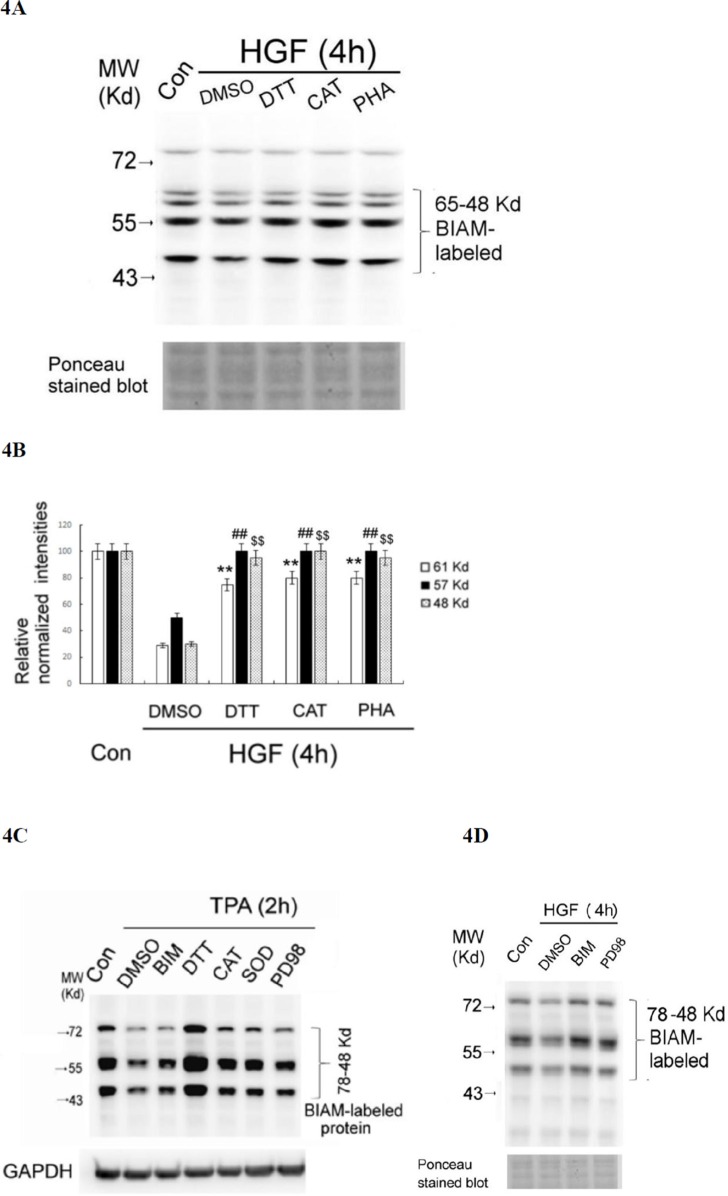
HGF- and TPA- induced reduction of SH-containing proteins were prevented by ROS scavengers and various inhibitors HepG2 were untreated (Con), treated with 25 nM HGF or HGF coupled with indicated inhibitors for 4 h (**A**, **D**), or treated with 50 nM TPA or TPA coupled with indicated inhibitors for 2 h **(C)**. After indicated times, the cell were harvested for affinity blots of BIAM-labeled proteins using avidin-HRP. Nonspecific bands in Ponceau stained blots (A, D) or GAPDH (C) were shown for normalizing the band intensities of redox sensitive proteins. **(B)** is the quantitative figure for (A). Relative intensities for the normalized redox sensitive proteins with indicated M.W. were estimated, taking the data of the untreated cells as 100. (**) (^##^) and (^$$^) represent statistical significance (Student's *t* test: *p* < 0.005; *N* = 3) for differences of the proteins with M.W. of 61, 57, 48 Kd, respectively, between the indicated HGF/inhibitor and the HGF/DMSO groups. (C) and (D) are representatives of two reproducible experiments.

To confirm that the decrease of SH-containing proteins was indicative of protein oxidation, we examined whether this event can be prevented by ROS scavengers and antioxidants. As was expected, the HGF-induced decrease of SH-containing proteins at 4 h can be prevented by co-treatment of HGF with DTT (0.5 mM) and CAT (500 units/ml) and SOD (500 units/ml) by about 50–60% (Figure 4A and 4B). As a positive control, PHA665752 (PHA) (20 nM), the inhibitor of c-Met, also effectively prevented HGF-induced global protein oxidation in a similar extent (Figure 4A and 4B). Similarly, the TPA-induced decrease of SH-containing proteins at 2 h can be greatly prevented by DTT (0.5 mM), CAT (500 units/ml) and SOD (500 units/ml) (Figure 4C). Notably, DTT not only prevented the decrease of SH-containing proteins induced by TPA at 4 h but also enhanced them as compared with those in the control group (Figure 4C), probably due to the high concentration of the DTT (which contain multiple SH- groups) used. In addition, the decreases of SH-containing proteins induced by HGF at 2 h (Figure 4D) and TPA at 4 h (Figure 4C) can also be significantly prevented by PD98059 (7.0 μM) and BIM (5.0 μM), inhibitors of ERK and PKC, respectively, known to be associated with ROS signaling [40, 41]. This implied that activations of both ERK and PKC were also required for ROS generation and protein oxidation induced by HGF and TPA. However, the underlying mechanisms need to be further explored.

### Identification of HSP60 and PDI as common redox sensitive proteins responsive to HGF and other ROS generators

**Figure 5:**
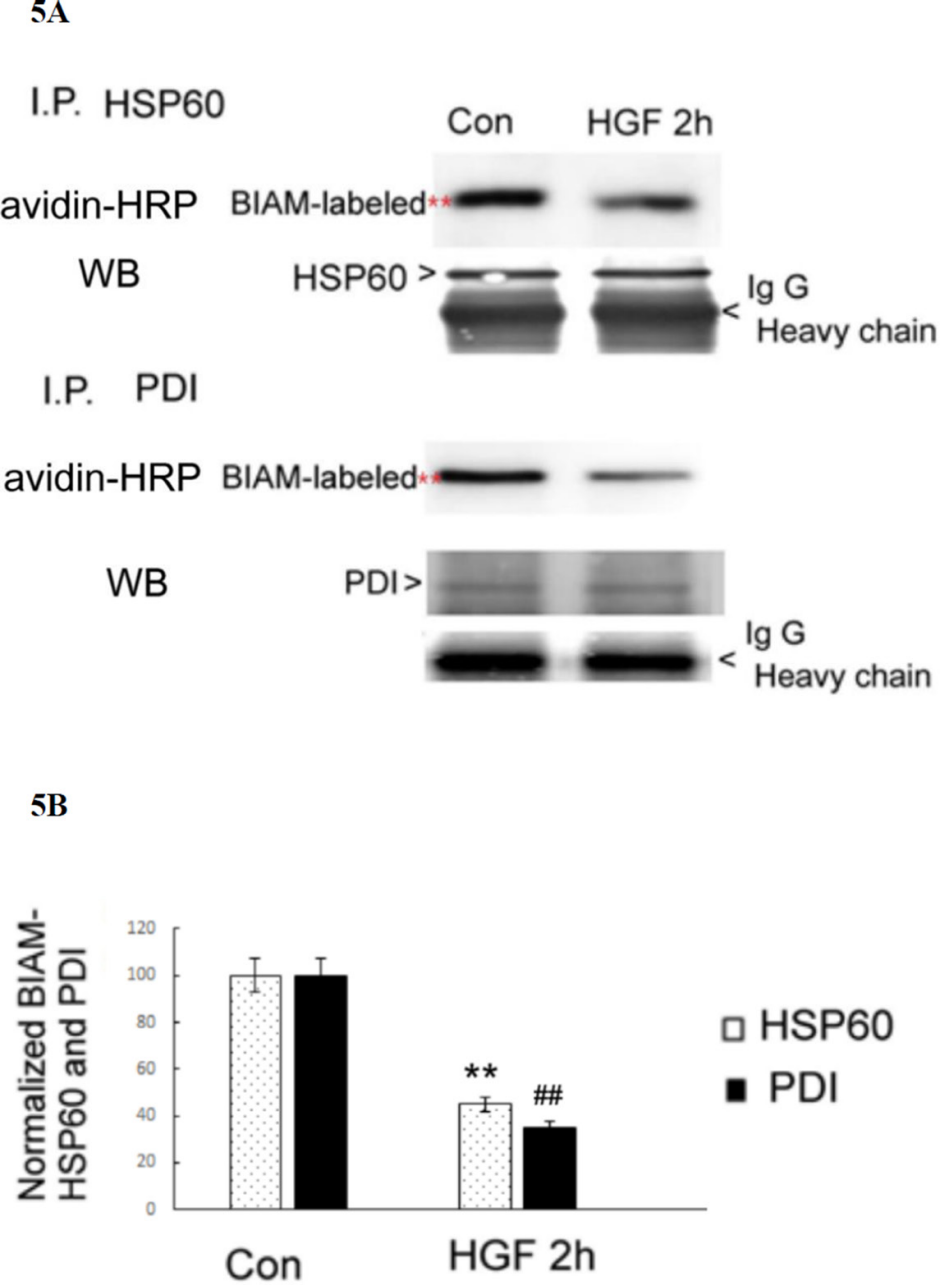
HGF suppressed SH-containing HSP60 and PDI in HepG2 (**A**) HepG2 were untreated (Con) or treated with 25 nM HGF for 2 h. Immunoprecipitation (IP) of HSP60 or PDI were performed followed by affinity blots using avidin-HRP for BIAM-labeled proteins. For the internal control, IP of HSP60 and PDI followed by Western blots for either protein were performed. IgG H (heavy chain of immunoglobin G) are indicated below each blots for additional IP control. The positions of BIAM-labeled HSP60 and PDI were indicated by red stars whereas those of HSP60, PDI and IgG H were indicated by arrow heads. **(B)** is the quantitative figure for (A). Relative intensities for the normalized BIAM-labeled HSP60 and PDI were calculated, taking the data of untreated cells as 100. (**) and (^##^) represent statistical significance (Student's *t p* < 0.005; *N* = 3) for intensity differences of BIAM-labeled HSP60 and PDI, respectively, between the HGF-treated and control groups.

To identify the ROS responsive proteins within the HGF- and TPA-induced signal pathway, the candidate proteins detected in the BIAM-labeling method were excised from the corresponding location in parallel coomassie blue-stained gels and identified by matrix-assisted laser desorption ionization-time of flight mass spectrometry (MALDI-TOF) analysis. The redox sensitive proteins identified in the HGF-treated HepG2 included heat shock protein 60 (HSP60) (61 KD), protein disulfide isomerase (PDI) (57 Kd), protein disulfide isomerase 3 (PDI3) (56.7 Kd), calreticulin (48 Kd) and tubulin (49 Kd). On the other hand, the redox sensitive proteins in TPA -treated HepG2 were HSP60 (61 Kd), PDI (57 Kd), alpha-enolase (ENOA) (56.7 Kd) and nuclease-sensitive element-binding protein 1 (YBOX1) (48 Kd). Similarly, the redox-sensitive proteins in PMS-treated HepG2 were HSP60 (61 Kd), PDI (57 Kd), PDIA3 (56.7 Kd) and tubulin (49 Kd). Among the proteins identified, HSP60 and PDI, which are classified as chaperones, can be found in all the three experimental groups. This suggested that they are the most essential redox sensitive proteins in HepG2 treated with all three agonists (i.e. HGF, TPA and PMS). To confirm whether HGF may induce oxidation of both chaperone proteins, the BIAM-labeled proteins were immunoprecipitated by antibodies of HSP60 or PDI followed by affinity blot analysis with avidin-HRP. As demonstrated in Figure 5A and 5B, the BIAM-labeled HSP60 and PDI were decreased by 56 and 65%, respectively, in cell lysates of HepG2 treated with 25 nM HGF for 2 h, compared with those of the untreated HepG2.

The BIAM-labeling method which detects the decrease of SH-containing protein is used for estimation of protein oxidization in an indirect way. To confirm the HGF-induced oxidation of both HSP 60 and PDI more directly, whether the putative oxidative products of both chaperons are elevated after HGF treatment are further examined. In general, the redox sensitive cysteine residues are easily converted to an unstable sulfenic form (RSOH), which can undergo further oxidation via disproportionation to sulfinic (RSO2H) species. Other cysteine modifications include nitrosylation (RSNO), glutathionylation (RSSG), or the formation of an inter- or intramolecular disulfide bond (RSSR) [26]. Specifically, the importance of reversible -S-glutathionylation of proteins (RSSG) in signal transduction under oxidative stress was highlighted recently [59]. As was expected, treatment of HGF for 1–2 h elevated -S-glutathionylated HSP60 to 4.1 and 3.1-fold followed by a decrease to 1.5 –fold at 4 h and rise again to 4.1–fold at 6 h, indicating a biphasic induction of -S-glutathionylation of HSP60 within this period (Supplementary Figure S1). On the other hand, TPA induced -S-glutathionylation of HSP60 within 1–2 h to 1.8–2.0 fold. However -S-glutathionylation of PDI was not elevated by treatment of both HGF and TPA (data not shown).

### HSP 60 and PDI were required for HGF-induced ERK phosphorylation and cell migration

**Figure 6:**
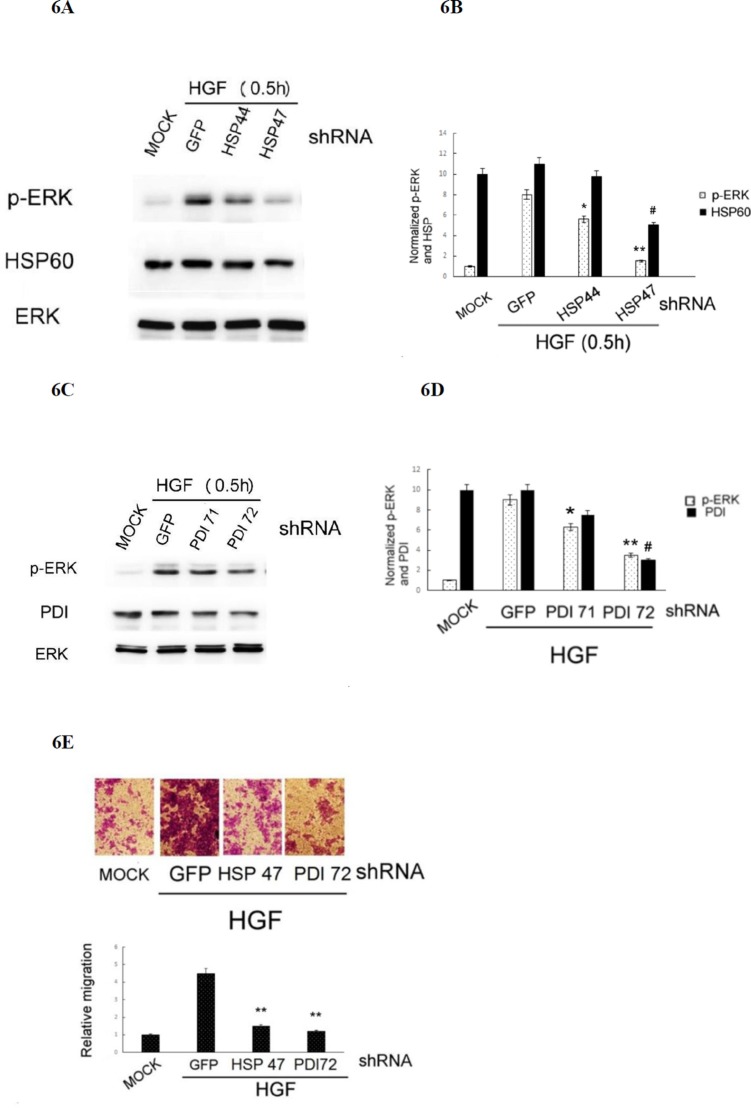
Depletion of HSP60 or PDI prevented HGF-induced ERK phosphorylation and HepG2 cell migration (**A**) HepG2 cells were transiently transfected with none (MOCK), GFP shRNA (as control shRNA) or various shRNA fragments of HSP60 (A) and PDI **(C)** for 36 h followed by treatment with 25 nM HGF for 30 min. Western blot of p-ERK (A) and (C), HSP60 (A) and PDI (C) were performed, using ERK for normalizing the band intensities. **(B)** and **(D)** are the quantitative figures for (A) and (C), respectively. Relative intensities for the normalized proteins were calculated, taking the data of MOCK samples as 1.0. (**), (*), (^##^) represent statistical significance (Student's *t p* < 0.005; *p* < 0. 05; *p* < 0.005, respectively *n* = 3) for intensity differences of p-ERK between the indicated shRNAs and the control GFP shRNA groups. **(E)** HepG2 cells were transiently transfected with none (MOCK), shRNA of GFP (as a control shRNA), HSP60 (HSP 47 plasmid) and PDI (PDI 72 plasmid) for 36 h followed by treatment with 25 nM HGF for 48 h. Transwell migration assay were performed. Pictures were taken under phase contrast microscope (200 *×* magnification) (upper panel) and the migrated cells were quantitated (lower panel). Relative migratory activity was calculated, taking the data of MOCK sample as 1.0. (**) represent statistical significance (Student's *t* test: *p* < 0.005, *N* = 3) for differences of relative migratory activity between the indicated shRNA and the GFP shRNA groups.

Whether HSP 60 and PDI are required for HGF-triggered ROS-ERK signaling and cell migration was further investigated by RNA interference using plasmids encoding shRNAs of HSP60 and PDI. As demonstrated in Figure 6A and 6B, transfection of the cells with HSP 47 plasmid encoding the HSP60 shRNA (the depletion efficiency for HSP60 was about 50%) blocked HGF-induced phosphorylation of ERK by 87%, as compared with that in the cell transfected with the control GFP shRNA. For comparison, transfection of the cell with HSP 44 plasmid encoding a less effective HSP60shRNA (with depletion efficiency of 10–15%) decreased HGF-induced phosphorylation of ERK by only 30%. Similarly, transfection of the cells with PDI 72 plasmid encoding the effective PDI shRNA (the depletion efficiency for PDI was about 70%) blocked HGF-induced phosphorylation of ERK by 53%, as compared with that in the cell transfected with the control GFP shRNA (Figure 6C and 6D). For comparison, transfection of the cells with PDI 71 plasmid encoding a less effective PDI shRNA (with depletion efficiency of 25%), suppressed HGF-induced phosphorylation of ERK by only 28%. Moreover, depletion of HSP60 and PDI in HepG2 by transfection of HSP 47 and PDI 72, the plasmids encoding the more effective HSP60 and PDI shRNAs, respectively, suppressed HGF-induced cell migration by 70–80% (Figure 6E). Collectively, these results indicated that both HSP60 and PDI were required for HGF-induced ERK activation and HepG2 cell migration.

### Identification of cysteines on HSP60 and PDI that are critical for HGF-induced redox signal and cell migration

**Figure 7:**
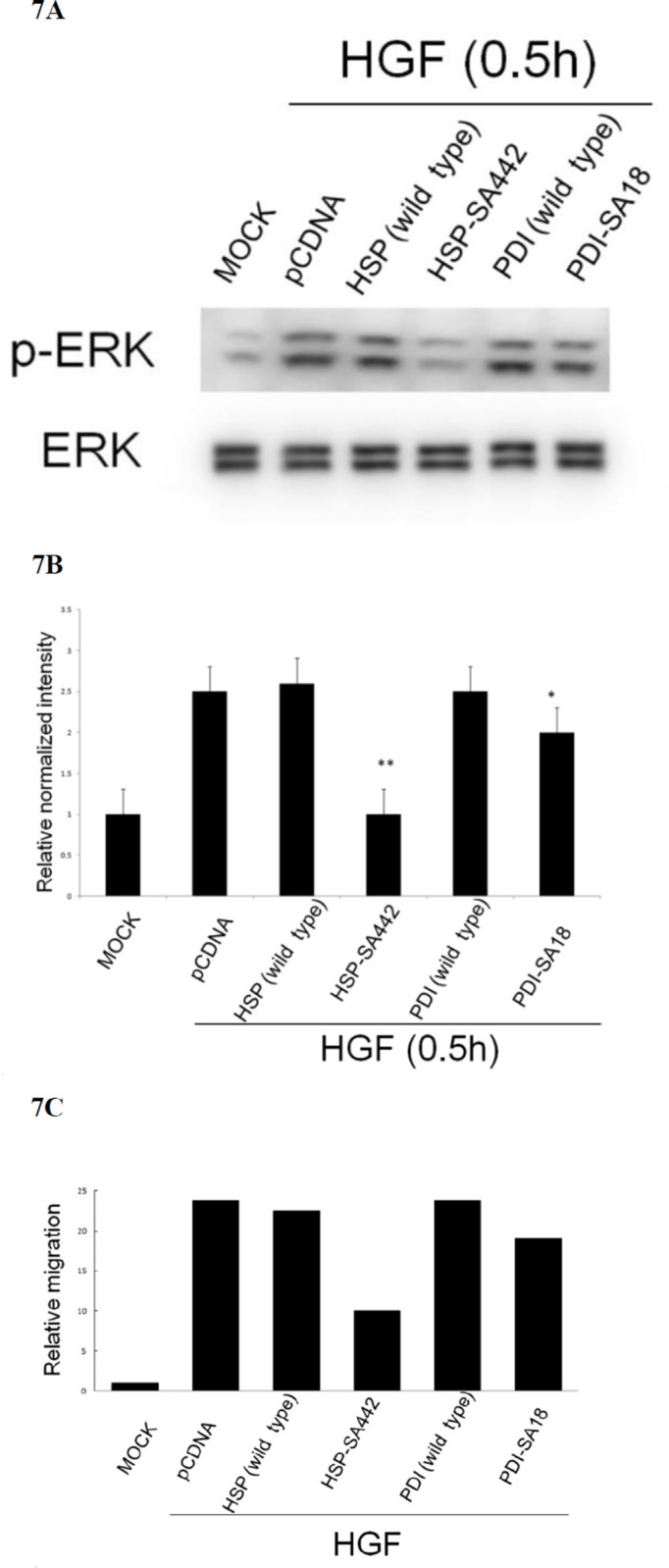
Alteration of cysteine on HSP60 prevented HGF-induced ERK phosphorylation and HepG2 cell migration HepG2 cells were transiently transfected with none (MOCK), pcDNA vector or wild type or cysteine mutant of HSP60 and PDI for 36 h followed by treatment with 25 nM HGF for 30 min **(A) (B)**, or 48 h **(C)**. Western blot of p-ERK (A) and transwell cell migration (C) were performed. In (A) ERK was used for normalizing the band intensities. (B) is the quantitative figures for (A). (**) represent statistical significance (Student's *t* test: *p* < 0.005, *N* = 3) (**) and (*) represent statistical significance (Student's *t* test: *p* < 0.005, and *p* < 0.05, respectively, *N* = 3) for differences of relative normalized intensity between HSP-SA442 (the HSP60 mutant) and wild type HSP60, and that between PDI-SA18 (the PDI mutant) and wild type PDI. In (C), relative migratory activity for each treatment was calculated as average of two reproducible result, taking the data of MOCK sample as 1.0.

We further examined the redox sensitive cysteine on both chaperones that are critical for HGF-induced ROS signal and cell migration. There are 3 cysteine residues (at codon 237, 442 and 447) on HSP60; and 7 (at codon 18, 71, 74, 418, 421, and 550) on PDI potentially involved in protein oxidation (Supplementary Figure S2). Site directed mutagenesis on HSP60 and PDI c-DNA sequences encoding cysteine was performed to obtain potential dominant negative mutants with change of cysteine to alanine (SA mutant) (Supplementary Figure S3 and S4). Due to the high GC content on both chaperones (which made the cloning of the mutant rather difficult), only S-A mutant of Cys 442 (the 3rd Cys codon) and Cys18 (the 1st Cys codon) of HSP60 and PDI, respectively, were obtained and denoted as HSP-SA442 and PDI-SA18. As demonstrated in Figure 7A and 7B, HGF-induced phosphorylation of ERK was totally abolished in HepG2 expressing HSP60-SA442, compared with that in the cell expressing wild type HSP60 or control vector. However, expression of PDI-SA18 only slightly prevented HGF-induced phosphorylation of ERK (by 18%). Consistently, transient expression of HSP60-SA442 greatly suppressed HGF-induced cell migration of HepG2 by 60% as compared with that in the cell expressing wild type HSP60 or control vector, whereas transient expression of PDI-SA18 exerted slight suppressive effects (Figure 7C). Collectively, cysteine442 on HSP60 but not cysteine18 on PDI was essential for HGF-induced ERK phosphorylation and cell migration.

### HSP60 is also a ROS sensitive protein required for HGF-induced ERK activation in HCC340

Whether HSP60 and PDI could perform as ROS sensitive proteins required for HGF-induced signal transduction was also investigated in another HCC, HCC340, a patient derived HCC cell line. Previous report has demonstrated HGF-induced cell migration of HCC340 via MAPK pathway [78]. By BIAM assay, significant decrease of two SH-containing proteins with molecular weight (M.W.) of about 60 and 50 KD was observed. The 60 Kd SH-containing proteins decreased by 50 and 45, 15, 52, and 60% in cells treated with HGF for 0.5, 1, 2, 4, and 6 h, respectively (Supplementary Figure S5A and S5B). Notably, the decrease of this protein was less prominent at 2 h compared with those at the earlier (1 and 2 h) and later (4 and 6 h) time points, thus implicating a biphasic pattern of decrease. On the other hand, the 50 Kd SH-containing proteins significantly decreased (by 20–25%) at 1 and 2 h followed by a slight decrease (by 10–15%) at 4 and 6 h (Supplementary Figure S5A and S5B). Further, the redox sensitive proteins with M.W. about 60 and 50 Kd were excised from the parallel coomassie blue-stained gels and forwarded to MALDI-TOF analysis. Interestingly, the candidate redox proteins identified were heat shock protein 60 (HSP60) (61 KD) and tubulin (49 Kd), in similar with those identified in HepG2. To validate HSP60 as a redox sensitive protein, the BIAM-labeled proteins was immunoprecipitated with HSP60 antibody followed by avidin affinity blot and Western blot of -S-glutathionylated proteins. As demonstrated in Supplementary Figure S6, the BIAM-labeled HSP60 decreased by 56% in cell lysates of HCC340 treated with 25 nM HGF at 6 h, compared with that at zero time point. Consistently, the -S-glutathionylated HSP60 increased by 2.2-fold at the same time point. Thus HSP60 was indeed the most critical ROS responsive protein in HCC340.

We further examined whether HSP60 was required for HGF-induced ERK signaling in HCC340. As demonstrated in Supplementary Figure S7A, transfection of the cells with HSP47 plasmid encoding the effective HSP60 shRNA blocked HGF-induced phosphorylation of ERK by 70–75%, as compared with that in the cell transfected with the control GFP or luciferase shRNA. HSP60 decreased in cell transfected with HSP60 shRNA by 75%, as compared with that of control GFP shRNA group, validating the depletion efficiency of HSP60 shRNA (Supplementary Figure S7B).

## DISCUSSION

### The role of protein disulfide isomerase and heat shock protein in tumor progression

In this study, we found HSP60 and PDI are the redox sensitive signal molecules mediating HGF-triggered signal pathway in HepG2. The similar observations for HSP60 was also recapitulated in HCC340. One interesting common feature of both PDI and HSP60 is that they were initially well known chaperone proteins in endoplasmic reticulum (ER) and mitochondria, respectively, and subsequently found to be associated with tumor progression. PDI (also known as GRp58 or ERp57) is a folding enzyme in ER with thiol oxidoreductase activity catalyzing disulfide bond formation, reduction, and isomerization of nascent substrates [60]. On the other hand, Hsp60 is a typical chaperone assisting protein folding in mitochondria, cytosol, cell surface and extracellular space [61]. PDI expression is higher in some cancers and involved in aggressive phenotypes of breast cancer [62], brain cancer [63] ovary cancer [64], prostate cancer [65], ovarian cancer [64] and HCC [66]. Also, HSP60 was involved in the metastatic changes of HCC, pancreatic carcinoma [67] and lung cancer [68]. Thus both PDI [68] and HSP60 [69] were regarded as promising markers and therapeutic targets for diagnosis, prognosis, prevention and treatment of various human cancers. However, the molecular mechanism for PDI and HSP60 to trigger tumor progression has not been fully established before.

### Implication of HSP60 and PDI as connectors for establishing ROS-ERK signaling

ROS signaling was known to be involved in several pathophysiological processes triggered by PDI and HSP60. Previously, both PDI and HSP60 were found to be oxidized in HepG2, responsible for cellular injury induced by alcohol [69]. Also, PDI-regulated protein-protein interactions was controlled via a redox mechanism [70]. On the other hand, PDI was required for NADPH oxidase-dependent ROS generation and Akt phosphorylation [71] and catalyzed the unfold protein response including ROS generation in ER. [72]. Also, HSP60 may strongly enhance the production of ROS in leukocyte stimulated by TPA [73]. Together, both PDI and HSP60 can be either upstream or down stream of ROS in various cellular processes. In our results, whereas HGF-induced ERK phosphorylation can be prevented by the antioxidant DTT (Figure 2), blockade of ERK activation decreased protein oxidation induced by both HGF and TPA (Figure 4A and 4C, respectively), implicating that ERK was either upstream or down stream of ROS. Since both PDI and HSP60 are required for ERK phosphorylation (Figure 6A–6D), it is very probable that they are also either upstream or down stream of ROS in HGF and TPA-triggered signal transduction.

### Involvement of cysteine on HSP60 in HGF-induced ROS signaling

We found the dominant negative SA mutant of HSP60, HSP-SA442, effectively prevented HGF-induced ERK phosphorylation and cell migration (Figure 7), validating the requirement of oxidative modification on HSP60 in these events. On the other hand, the SA mutant of PDI, PDI-SA18, didn't exhibit significant suppressive effect. Thus far, the other SA mutants of PDI have not been obtained for testing the dominant negative activity, whether oxidative modification of PDI was involved in HGF-induced ERK phosphorylation and cell migration are not certain.

## CONCLUSION

Taken together, whereas HGF could induce ROS generation for ERK signaling, cell migration and I.M. of HepG2, HSP 60 and PDI were possibly required for linking ROS to ERK. Moreover, the critical cysteine residue on HSP60 responsible for redox signaling and cell migration induced by HGF was identified. In addition, HSP60 is also a redox sensitive protein required for HGF-induced ERK activation in HCC340. It can be anticipated that HSP60 is a promising therapeutic target for preventing HGF-induced HCC progression mediated by ROS.

## MATERIALS AND METHODS

### Cell lines and chemicals

Conventional hepatoma cells HepG2 is available in this Lab. HGF and TPA were purchased from Sigma (St. Louis, MO, US). 2′, 7′-Dichlorodihydrofluorescin diacetate, catalase, dithiotheritol, diphenyleneiodonium chloride, NSC23766, PD98059, PHA665752 and phenazine methosulfate were purchased from Calbiochem (Darmstadt, Germany). Antibodies for PDI, HSP60, p-ERK, ERK and GAPDH were from Santa Cruz (CA, US). Antibody for -S-glutathione was from Abcam (Cambridge, U.K.)

### Transwell migration assay

Migration assays were done using a 24-well transwell migration insert (Nalge Nunc International, Rochester, NY). Cells were seeded (5 *×* 10^4^ per upper chamber) in complete medium for 24 h followed by appropriate treatments. Cells that have migrated to the underside of the insert membrane were stained with 0.3% crystal violet. The cells in the upper side of the insert membrane were rubbed with a cotton swab. The migrated cells on the underside were pictured under a 200 *×* magnification field followed by quantitation using Image J. Software.

### Immunoprecipitation and western blot

Immunoprecipitation and Western blot [76] analysis were performed according to our previous reports. The band intensities on the blots were quantified with Image J. Software.

### Flow cytometric analysis for ROS generation

ROS assay was performed as described in our previous report [40]. The cells were seeded in 24-well plates, treated with tested agents for appropriate time. Then DCF-DA was applied and incubated for another 20 min at 37°C. Subsequently, the cells were washed twice with PBS and harvested for flow cytometric detection, using FACSort (Becton-Dickinson, Rutherford, NJ) with a 488-nm excitation beam. The signals were obtained using a 530-nm bandpass filter (FL-1 channel) for DCF. Each determination is based on the mean fluorescence intensity of 5,000 cells.

### Establishment of intrahepatic metastasis in SCID mice

The intrahepatic metastasis of HepG2 was established using Nod SCID mice according to previous reports [40, 41, 54]. Exponentially growing HepG2 cells were harvested from subconfluent cultures. The cells suspended in 100 μl DMEM were directly inoculated (using a 27-gauge needle and a 1-mL syringe) into the subserosa of middle liver lobe of mice (3.0 *×* 10^5^/mouse) under anesthesia with ketamine.

Starting on day 1 after inoculation of tumor cell, the mice injected with hepatoma cells were randomly divided into several groups for intraperitoneal administration twice a week with various agents such as DMEM only, HGF only, HGF plus various inhibitors. After 3–4 months, the animals were sacrificed after anesthesia with ketamine to observe tumor formation in liver and other organs. Nodules with diameter more than 0.1–0.2 cm observed on left or right lobes were recognized as 2nd tumor foci. Intrahepatic metastasis were assigned if at least two 2nd tumor foci can be observed in left and/or right lobes of liver of the treated mouse. During animal experiment, the regulations relevant to the care and use of laboratory animals were followed. This was approved by the Institutional Animal Care and Use Committee (IACUC) at the Tzu Chi Hospital.

### Determination of thiol modification

After required treatments, the cells were lysed with a buffer containing 0.5% (v/v) Triton X-100, 1 mM EDTA, protease inhibitors, and 100 μM *N*-(biotinoyl)-*N*′-(iodoacetyl)-ethylenediamine (BIAM), which labels reduced protein thiols. After separation by PAGE and electroblotting, BIAM-labeled proteins were detected using horseradish peroxidase-conjugated streptavidin.

### Gel purification of proteins and MS/MS analysis

The redox sensitive proteins were gel-purified using Coomassie in-gel digestion method (EMBL protocol). Briefly, after the interested bands were excised from Coomassie blue stained gel, they were washed sequentially with 50/50 acetonitrile (ACN)/ddH_2_O and 100 mM ammonium bicarbonate. The washed gels were dried in a speed vacuum followed by digestion with trypsin for overnight at 37°C. The proteins were eluted by addition of 50/50 ACN/5% formic acid and treated with DTT (1 mM). The eluates were then completely dried in speed vacuum and reconstituted in 15 μL 5% formic acid for mass spectrometric analysis. The peptide mixtures (come from in-gel digestion) were forwarded to Matrix-assisted laser desorption ionization-time of flight mass spectrometry (MALDI-TOF) analysis. The oxidized proteins were predicted and identified based on its Mass profile by Biowork search engine with adequate bioinformation.

### Transient depletion of PDI and HSP60

Plasmid DNA each encoding shRNAs targeting different mRNA regions of PDI or HSP60 were obtained from RNAi Core Laboratory in Academia Sinica, Taiwan. the cells were transfected with 25 nM of indicated shRNA for 48 h by lipofetamin, according to the manufacture's protocol. The knock down efficiency of indicated molecules was verified by Western blot as demonstrated in the text.

### Construction and expression of PDI and HSP60 mutants defective on cysteine coding regions

The indicated cysteine coding regions (TGT, TGC) on PDI and HSP60 were changed into that for alanine (GCX) by KOD-plus-mutagenesis kit (TOYOBO, Japan) (Supplementary Figure S3 and S4). The mutated plasmids were transformed into E.coli (DH5) followed by selection with Ampicillin. The wild type and mutant plasmids were transfected into HepG2 using lipofectmin. The expression of wild type and site-directed mutants of both chaperones has been validated by Western blot using Ab against each molecule. Since the endogenous HSP60 and PDI are rather high, the exogenous HSP60 and PDI (either wild type or mutant) in the cells transfected with each expressing plasmid increased by only10–20% compared with that in the cell transfected with pcDNA3 vector (data not shown).

### Statistical analysis

Paired Student's *t*-tests were performed to statistically analyze the differences of ROS generation, quantitative migration and band intensity on Western blot between the indicated groups. For animal experiment, observations and evaluations of the growth of primary tumor in middle lobe and intrahepatic metastasis toward left or right liver lobe were statistically analyzed using the Statistical Package for Social Sciences (SPSS, version 17.0) involved descriptive statistics (for single group analysis).

## SUPPLEMENTARY MATERIALS FIGURES



## Abbreviations

HCC: hepatocellular carcinoma
HGF: hepatocyte growth factor
TPA: 12-O-tetradecanoyl-phorbol-13-acetate
EGF: epidermal growth factor
TGFβ: transforming growth factor-β
PMS: phenazine methosulfate
EMT: epithelial mesenchymal transition
ROS: reactive oxygen species
HSP60: heat shock protein 60
PDI: protein disulfide isomerase
MAPK: mitogen activated protein kinase
ERK: extracellular signal-regulated kinases
PKC: protein kinase C
BIAM: N-(biotinoyl)-N′-(iodoacetyl)- ethylenediamine
DCFH-DA: 2′,7′- dichlorodihydrofluorescein diacetate
PD: PD98059
NSC: NSC23766
CAT: catalase
SOD: superoxide dismutase
DTT: dithiotheritol
BIM: Bisindolylmaleimides.
