# Supplementary material for: Oxidation of heat shock protein 60 and protein disulfide isomerase activates ERK and migration of human hepatocellular carcinoma HepG2
Source: Oncotarget. 2016 Jan 31;7(10):11067–82. doi: 10.18632/oncotarget.7093 (PMC4905458; doi:10.18632/oncotarget.7093)
Supplement: Supplementary file 1 [file oncotarget-07-11067-s001.pdf]

Oxidation of heat shock protein 60 and protein disulfide isomerase activates ERK and migration of human hepatocellular carcinoma HepG2

Supplementary Materials

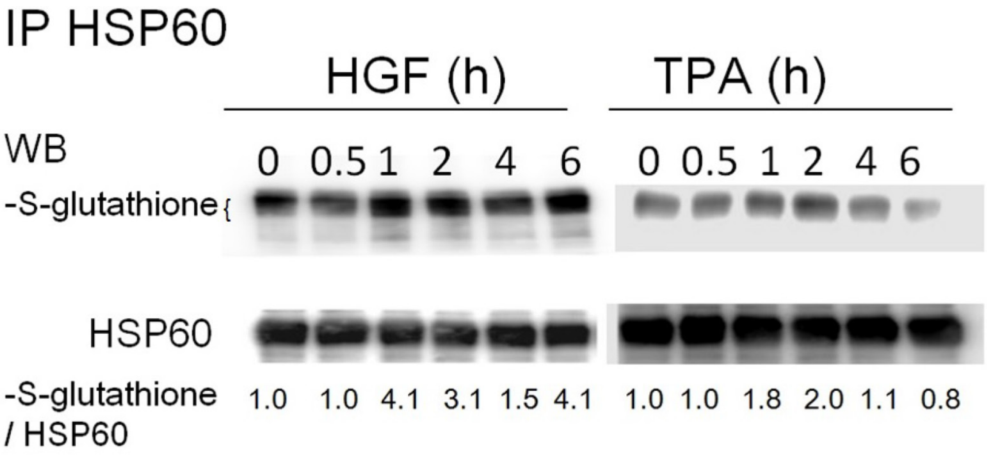

Supplementary Figure S1: Induction of glutathionated HSP60 by HGF and TPA. HepG2 cells were untreated (Con) or treated with 25 nM HGF for indicated times. Immunoprecipitation (IP) of HSP60 followed by Western blot for –S glutathionated proteins. For the internal control, IP of HSP60 followed by Western blots for glutathionated proteins were performed. The positions of glutathionated HSP60 were indicated by {. The numbers shown below were average of two reproducible relative intensities of normalized glutathionated HSP60, taking the data of the untreated as 1.0.

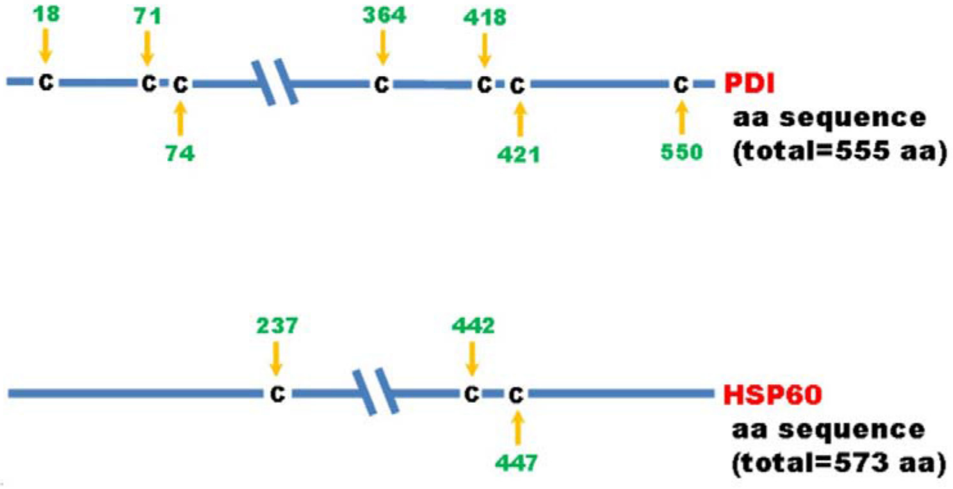

Supplementary Figure S2: Positions of Cys on PDI and HSP60. Schematic maps indicating the position of cysteine (C) on PDI (upper) and HSP60 (lower). The numbers indicated above is the sequence number of the indicated cysteine.

(PDI-1625-wild **forward primer)** **Start codn**

ATCCC**CTTCTGG**CCCCCTTATCTGCCCCCGCCCCACGCGCCCTGGCAGCACC**ATG**AGCCGCC

AGCTTCTGCCTGTACTGCTGCTGCTGCTGCTCAGGGCTTCG **【TGC⇒GCC】** site of **mutagenesis**

CCATGGGGTCAGGAACAGGGAGCGAGGAGCCCCTCGGAGGAGCCTCCAGAGGAGGAAAT

CCCCAAGGAGGATGGGATCTTGGTGCTGAGCCGCCACACCCTGGGCCTGGCCCTGCGGGA

GCACCCTGCCCTGCTGGTGGAATTCTATGCCCCGTGG**TGT**GGGCAC**TGC**CAGGCCCTGGCC

CCCGAGTACAGCAAGGCAGCTGCCGTGCTCGCGGCCGAGTCAATGGTGGTCACGCTGGCC

AAGGTGGATGGGCCCGCGCAGCGCGAGCTGGCTGAGGAGTTTGGTGTGACGGAGTACCCT

ACGCTCAAGTTCTTCCGCAATGGGAACCGCACGCACCCGGAGGAGTACACAGGACCACGG

GACGCTGAGGGCATTGCCGAGTGGCTGCGACGGCGGGTGGGGCCCAGTGCCATGCGGCT

GGAGGACGAGGCGGCCGCCCAGGCGCTGATCGGTGGCCGGGACCTAGTGGTCATTGGCTT

CTTCCAGGACCTGCAGGACGAGGACGTGGCCACCTTCTTGGCCTTGGCCCAGGACGCCCTG

GACATGACCTTTGGCCTCACAGACCGGCCGCGGCTCTTTCAGCAGTTTGGCCTCACCAAGGA

CACTGTGGTTCTCTTCAAGAAGTTTGATGAGGGGCGGGCAGACTTCCCCGTGGACGAGGAG

CTTGGCCTGGACCTGGGGGATCTGTCGCGCTTCCTGGTCACACACAGCATGCGCCTGGTCA

CGGAGTTCAACAGCCAGACGTCTGCCAAGATCTTCGCGGCCAGGATCCTCAACCACCTGCT

GCTGTTTGTCAACCAGACGCTGGCTGCGCACCGGGAGCTCCTAGCGGGCTTTGGGGAGGC

AGCTCCCCGCTTCCGGGGGCAGGTGCTGTTCGTGGTGGTGGACGTGGCGGCCGACAATGA

GCACGTGCTGCAGTACTTTGGACTCAAGGCTGAGGCAGCCCCCACTCTGCGCTTGGTCAAC

CTTGAAACCACTAAGAAGTATGCGCCTGTGGATGGGGGCCCTGTCACCGCAGCGTCCATCA

CTGCTTTC**TGC**CATGCAGTCCTCAACGGCCAAGTCAAGCCCTATCTCCTGAGCCAGGAGATA

CCCCCTGATTGGGATCAGCGGCCAGTTAAGACCCTCGTGGGCAAGAATTTTGAGCAGGTGG

CTTTTGACGAAACCAAGAATGTGTTTGTCAAGTTCTATGCCCCGTGG**TGC**ACCCAC**TGC**AAG

GAGATGGCCCCTGCCTGGGAGGCATTGGCTGAGAAGTACCAAGACCACGAGGACATCATC

ATTGCTGAGCTGGATGCCACGGCCAACGAGCTGGATGCCTTCGCTGTGCACGGCTTCCCTA

CTCTCAAGTACTTCCCAGCAGGGCCAGGTCGGAAGGTGATTGAATACAAAAGCACCAGGGA

CCTGGAGACTTTCTCCAAGTTCCTGGACAACGGGGGCGTGCTGCCCACGGAGGAGCCCCCG

GAGGAGCCAGCAGCCCCGTTCCCGGAGCCACCGGCCAACTCCACTATGGGGTCCAAGGAG

GAACTG**TAG** CTC GAG

Stop codn

Supplementary Figure S3: Site directed mutagenesis of Cys on PDI. Full genomic sequence of PDI containing proximal promoter region. The positions of start and stop codon are marked with blue. The sequence encoding Cysteine (TGC or TGT) are underlined and marked with yellow. There are 6 cystein codons on PDI. The 1st cystein codon marked with red was changed from TGC (Cys) to GCC (alanine) using KOD-plus-mutagenesis kit (TOYOBO, Japan). The template used was pPDI-1625 (wild type PDI genomic sequence inseted in pcDNA3.1 , inset size: 1625 bp)

«forward primer: GCC CCA TGG GGT CAG GAA CAG» G GAA CAG»

«reverse primer; CGA AGC CCT GAG CAG CAG»

(melting tempratur: 60°C).

Start

AACCTGGGCTGTTAAGCTTGGTACCTCGCGAATGCATCTAGATT**ATG**CTTCGGTTACCCACA
GTCTTTCGCCAGATGAGACCGGTGTCCAGGGTACTGGCTCCTCATCTCACTCGGGCTTATGC
CAAAGATGTAAAATTTGGTGCAGATGCCCGAGCCTTAATGCTTCAAGGTGTAGACCTTTTAG
CCGATGCTGTGGCCGTTACAATGGGGCCAAAGGGAAGAACAGTGATTATTGAGCAGAGTT
GGGGAAGTCCCAAAGTAACAAAAGATGGTGTGACTGTTGCAAAGTCAATTGACTTAAAAGA
TAAATACAAAAACATTGGAGCTAAACTTGTTCAAGATGTTGCCAATAACACAAATGAAGAA
GCTGGGGATGGCACTACCACTGCTACTGTACTGGCACGCTCTATAGCCAAGGAAGGCTTCG
AGAAGATTAGCAAAGGTGCTAATCCAGTGGAAATCAGGAGAGGTGTGATGTTAGCTGTTG
ATGCTGTAATTGCTGAACTTAAAAAGCAGTCTAAACCTGTGACCACCCCTGAAGAAATTGCA
CAGGTTGCTACGATTTCTGCAAACGGAGACAAAGAAATTGGCAATATCATCTCTGATGCAAT
GAAAAAAGTTGGAAGAAAGGGTGTCATCACAGTAAAGGATGGAAAAACACTGAATGATGA
ATTAGAAATTATTGAAGGCATGAAGTTTGATCGAGGCTATATTTCTCCATACTTTATTAATAC
ATCAAAAGGTCAGAAA**TGT**GAATTCCAGGATGCCTATGTTCTGTTGAGTGAAAAGAAAATT
TCTAGTATCCAGTCCATTGTACCTGCTCTTGAAATTGCCAATGCTCACCGTAAGCCTTTGGTC
ATAATCGCTGAAGATGTTGATGGAGAAGCTCTAAGTACACTCGTCTTGAATAGGCTAAAGG
TTGGTCTTCAGGTTGTGGCAGTCAAGGCTCCAGGGTTTGGTGACAATAGAAAGAACCAGCT
TAAAGATATGGCTATTGCTACTGGTGGTGCAGTGTTTGGAGAAGAGGGATTGACCCTGAAT
CTTGAAGACGTTCAGCCTCATGACTTAGGAAAAGTTGGAGAGGTCATTGTGACCAAAGACG
ATGCCATGCTCTTAAAAGGAAAAGGTGACAAGGCTCAAATTGAAAAACGTATTCAAGAAAT
CATTGAGCAGTTAGATGTCACAACTAGTGAATATGAAAAGGAAAAACTGAATGAACGGCTT
GCAAAACTTTCAGATGGAGTGGCTGTGCTGAAGGTTGGTGGGACAAGTGATGTTGAAGTG
AATGAAAAGAAAGACAGAGTTACAGATGCCCTTAATGCTACAAGAGCTGCTGTTGAAGAAG
GCATTGTTTTGGGAGGGGGT**TGT**GCCCTCCTTCGA **【TGC ⇒GCC】**
ATTCCAGCCTTGGACTCATT

site of **mutagenesis**

GACTCCAGCTAATGAAGATCAAAAAATTGGTATAGAAATTATTAAAAGAACACTCAAAATTC
CAGCAATGACCATTGCTAAGAATGCAGGTGTTGAAGGATCTTTGATAGTTGAGAAAATTAT
GCAAAGTTCCTCAGAAGTTGGTTATGATGCTATGGCTGGAGATTTTGTGAATATGGTGGAA
AAAGGAATCATTGACCCAACAAAGGTTGTGAGAACTGCTTTATTGGATGCTGCTGGTGTGG
CCTCTCTGTTAACTACAGCAGAAGTTGTAGTCACAGAAATTCCTAAAGAAGAGAAGGACCCT
GGAATGGGTGCAATGGGTGGAATGGGAGGTGGTATGGGAGGTGGCATGTTCTA**ACT**CTC
GAG

Stop

Supplementary Figure S4: Site directed mutagenesis of Cys on HSP60. Full genomic sequence of HSP60 containing proximal promoter region. The positions of marked with start and stop codon are marked with blue. The sequence encoding Cysteine (TGC or TGT) are underlined and marked with yellow. The Cysteine sequence that is mutated to Alanine (GCC) is also marked with Red. There are 3 cystein codons on HSP60. The third cystein codon marked with red was changed from TGC (Cys) to GCC (alanine) using KOD-plus-mutagenesis kit (TOYOBO, Japan). The template used was pHSP-1724 (wild type HSP60 genomic sequence inseted in pcDNA3.1 , inset size: 1724 bp)

«forward primer: **GCC** ATT **CCA GCC TTG GAC TCA**»

«reverse primer: **TCG AAG GAG GGC ACA ACC**»

(melting temperature: 55°C).

A

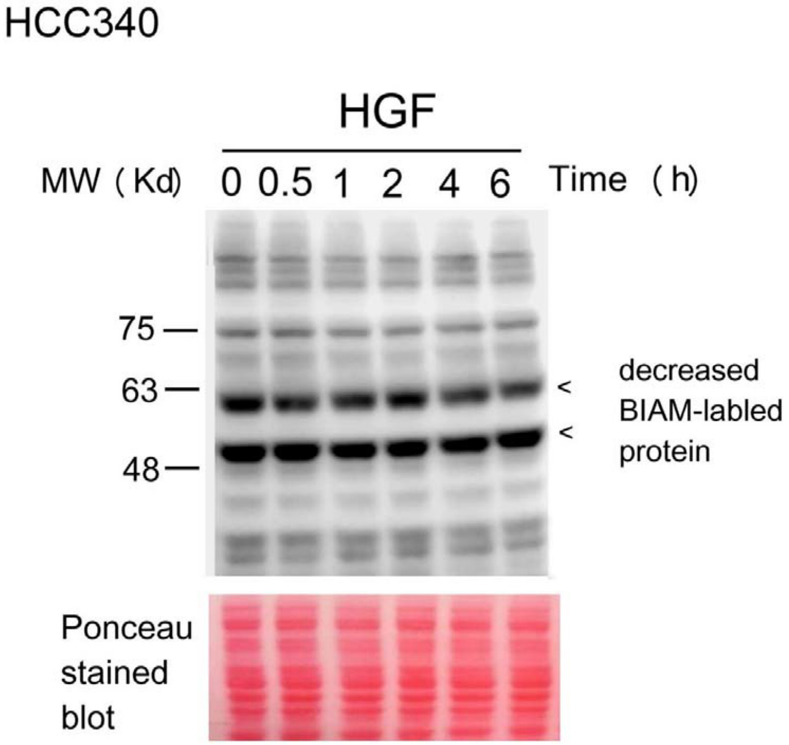

B

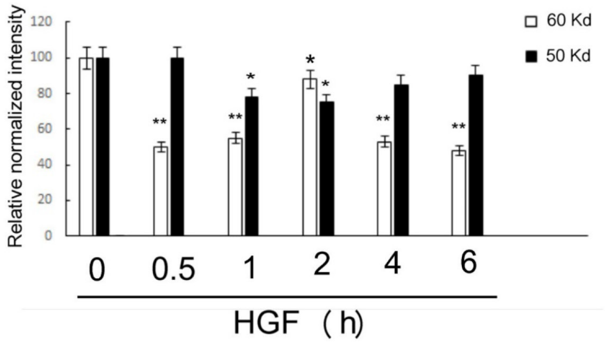

Supplementary Figure S5: HGF decreased multiple SH-containing proteins in HCC340. (**A**) HCC340 cells were treated with 25 nM HGF for indicated times. Affinity blots of BIAM-labeled proteins were performed using avidin-HRP. The position of BIAM-labeled proteins decreased in the treated samples were indicated by arrow heads. Ponceau stained blots were shown for normalizing the band intensities of redox sensitive proteins. (**B**) is the quantitative figure for (A). Relative intensities for the normalized redox sensitive proteins with indicated M.W. was estimated, taking the data of the untreated cells as 100. (**) and (*) represent statistical significance (Student's *t* test: $p < 0.005$ and $p < 0.05$, respectively; $N = 3$) for differences of the proteins with specific M.W. between the indicated sample and the zero time point.

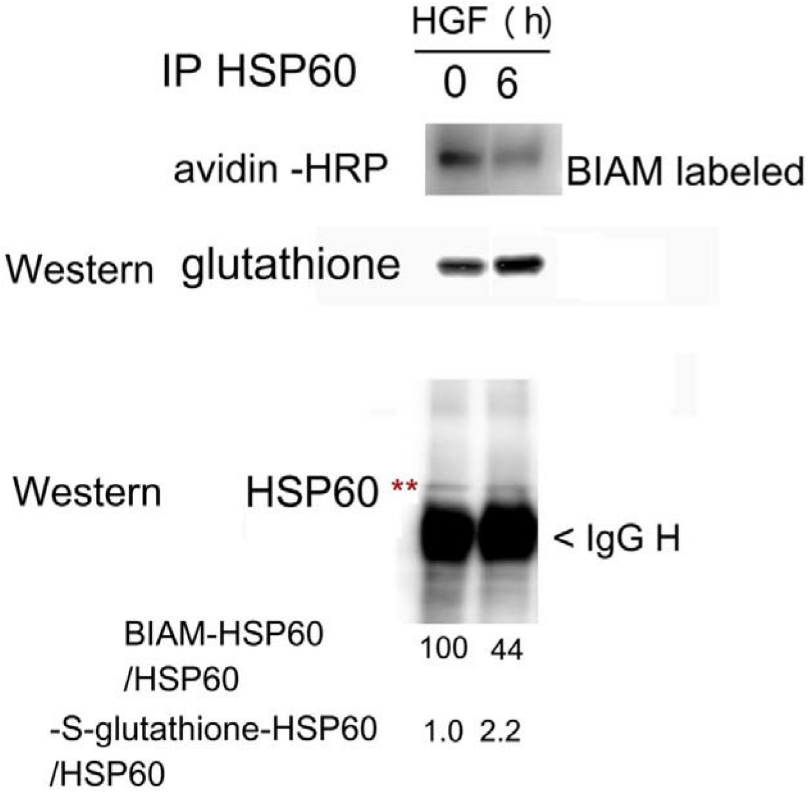

Supplementary Figure S6: HGF suppressed SH-containing HSP60 and elevated glutathionated HSP60 in HCC340. HCC340 was treated with 25 nM HGF for indicated times. Immunoprecipitation (IP) of HSP60 was performed followed by affinity blot using avidin-HRP for BIAM-labeled proteins (upper panel) and Western blot for –S– glutathionated proteins (middle panel). For the internal control, IP of HSP60 followed by Western blots of HSP 60 was performed (lower panel). IgG H (heavy chain of immunoglobin G) are indicated below the blots for additional IP control. The position of HSP60 was indicated by red stars (**) whereas that of heavy chain of immunoglobin G (IgG H) was indicated by arrow head. The numbers below are the relative ratios of BIAM-labeled HSP60 or –S- glutathionated HSP60 *vs* total HSP60, taking data of time zero as 100% and 1.0 respectively. The data shown was average of two experiments.

A

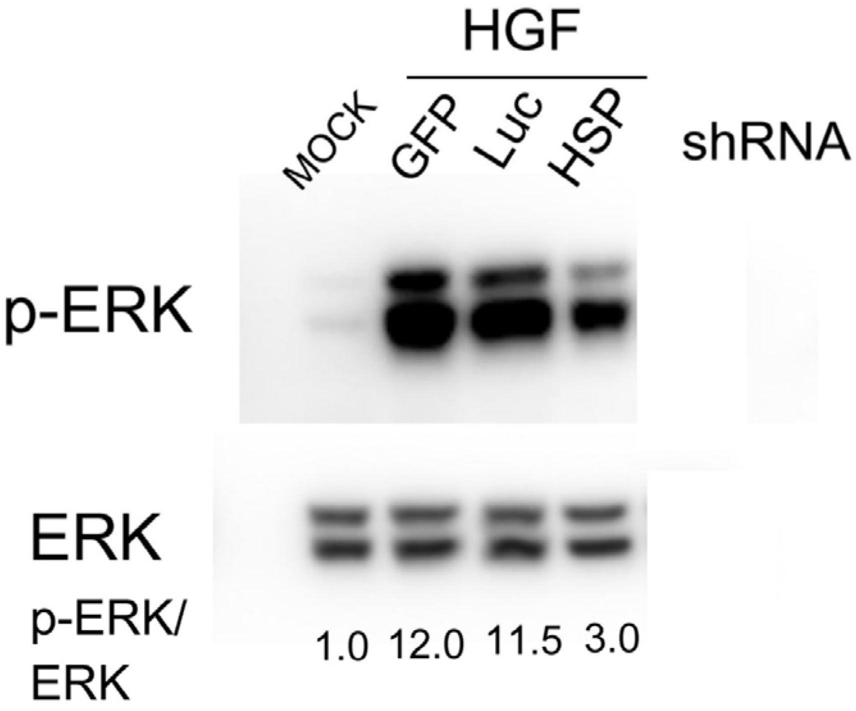

B

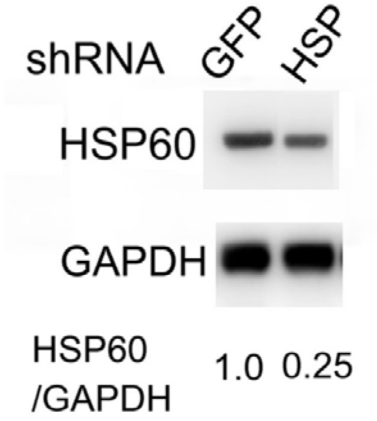

Supplementary Figure S7: Depletion of HSP60 prevented HGF-induced ERK phosphorylation in HCC340. (**A**) HCC340 cells was transiently transfected with none (MOCK), GFP and luciferase (Luc) shRNAs (as control shRNAs) or effective shRNAs of HSP60 (HSP47) for 36 h followed by treatment with 25 nM HGF for 30 min. Western blot of p-ERK was performed, using ERK for normalizing the band intensities. The numbers shown below are relative intensities for the normalized proteins (p-ERK/ERK), taking the data of MOCK samples as 1.0. The data shown were average of 3 experiments. (**B**) HCC340 cells was transiently transfected with GFP or effective shRNA of HSP60 (HSP47) for 48 h. Western blot of HSP60 was performed, using GAPDH as internal control. The numbers shown below are relative intensities for the normalized proteins (HSP60/GAPDH), taking the data of GFP samples as 1.0. The data shown were average of 2 experiments.
